# Surveillance of influenza viruses circulating from 2017/2018 to 2023/2024 seasons in Veneto Region, North-East Italy

**DOI:** 10.1186/s12985-025-02723-9

**Published:** 2025-04-24

**Authors:** Giuseppe Di Pietra, Denis Munegato, Chiara Poletto, Valeria Conciatori, Sarah Di Sopra, Elisa Franchin, Ignazio Castagliuolo, Cristiano Salata, Claudia Del Vecchio

**Affiliations:** 1 Department of Molecular Medicine, University of Padua, Via Gabelli 63, 35121 Padua, Italy; 2 Microbiology and Virology Diagnostic Unit, Padua University Hospital, Via Giustiniani 2, 35128 Padua, Italy

**Keywords:** Influenza virus, Influenza A, Influenza B, Surveillance, Epidemics, Veneto region

## Abstract

**Background:**

In Italy, influenza viruses typically circulate from October to April, causing seasonal epidemics. The pattern of influenza virus circulation varies each season regarding the timing of the first case notification, period of circulation, and predominant influenza virus types and subtypes.

**Methods:**

This analysis used comprehensive data from the national influenza surveillance network for the 2017/2018 to 2023/2024 influenza seasons in the Veneto Region. Influenza A (IAV) and B (IBV) viruses were detected and subtyped using real-time reverse transcriptase-polymerase chain reaction assays.

**Results:**

Of 21,180 oropharyngeal swabs collected from 2017 to 2024, 4,325 (20.42%) were positive for influenza viruses. IAV accounted for 78.68% of positive cases overall, representing more than 65% of cases in every season except 2017/2018 (26.72%). Both A(H1N1)pdm09 and A(H3N2) subtypes were detected in all seasons with varying proportions. IBV represented 21.32% of all positive cases, with Victoria and Yamagata lineages detected simultaneously during the 2017/2018 season. No Yamagata lineage was detected after the 2018/2019 season, and no IBV cases were detected in the 2021/2022 season. In almost all seasons, influenza virus circulation was more significant in adults, especially those 65 years and older, than in children.

**Conclusions:**

In the Veneto Region, influenza virus circulation varied considerably from 2017/2018 to 2023/2024. In the 2020/2021 season, no influenza-positive samples were detected due to circulation of SARS-CoV-2 and related countermeasures. IAVs were the predominant type in most seasons, while IBVs made a limited contribution to the overall disease burden.

## Introduction

Influenza is an acute respiratory illness caused by influenza viruses, spreading through droplets, aerosols, direct contact, or indirect contact with contaminated surfaces [1–3]. Clinical symptoms appear suddenly after around two days of incubation period and include fever, sore throat, cough, chills, headache, myalgia, rhinosinusitis, weakness, fatigue, and general malaise. Fever usually subsides within 3–7 days, though some infections are asymptomatic. According to the World Health Organization (WHO), there are 1 billion seasonal influenza cases annually, with 3–5 million severe cases and 290,000 to 650,000 deaths [4, 5]. Mortality is mainly due to respiratory complications, particularly in people over 65 years and those with high-risk conditions like asthma, heart disease, chronic kidney failure, or diabetes. Extra-respiratory complications can also occur at cardiovascular and neurological levels [6, 7]. Measures to counteract influenza spread include primary prevention with trivalent or quadrivalent vaccines and antiviral drugs [8–10].

Two species of Influenza viruses are clinically relevant in humans: Influenza A viruses (IAVs) and Influenza B viruses (IBVs). IAVs are further divided into many subtypes, specially A(H1N1)pdm09 and A(H3N2), which are involved in seasonal flu epidemics. IBVs are divided into two lineages: B/Victoria and B/Yamagata [11–14].

The high pandemic risk associated with zoonotic IAVs and the widespread annual influenza transmission led the WHO to establish an influenza surveillance network in the 1950s. This platform reports and analyzes influenza data shared through FluNet and FluID platforms by the Global Influenza Surveillance and Response System (GISRS) and national epidemiological institutions [15].

In Italy, surveillance has been active since the 1999/2000 season, integrating data from a network of primary care physicians, pediatricians, and hospital doctors through Regional Reference Laboratories for Respiratory Viruses coordinated by the National Institute of Health (Istituto Superiore di Sanità—ISS). The InfluNet network operated until the 2022/2023 season, and from 2023/2024, the new RespiVirNet network includes the surveillance of other respiratory viruses: respiratory syncytial virus, rhinovirus, parainfluenza viruses, adenovirus, bocavirus, metapneumovirus, SARS-CoV-2 and other human coronaviruses.

This study aims to describe the virological data collected in the Veneto Region, the fourth most populous Region in Italy, during the influenza seasons between 2017 and 2024, highlighting differences in virus circulation between the pre- and post-COVID-19 pandemic periods.

## Methods

### Study design

This study describes the circulation of IAVs and IBVs over seven consecutive influenza seasons from 2017/2018 to 2023/2024. Data were collected at the Microbiology and Virology unit of the Padua University Hospital, the Regional Reference Center for Influenza and other Respiratory infections, part of the Italian influenza surveillance network RespiVirNet. Oropharyngeal swabs were collected from each patient with Influenza-Like Illness (ILI), defined by the WHO as an acute respiratory infection with clinical manifestations such as fever and coughing, with onset within the last 10 days.

### Laboratory analysis

Laboratory analyses for influenza virus detection were conducted using an In-House Real-Time RT-PCR system until the 2020/2021 season. Briefly, total nucleic acids were purified from 200 μl of nasopharyngeal swab samples and eluted in a final volume of 100 μl by using a MagNA Pure 96 System (Roche Applied Sciences). Detection of Influenza viruses RNA were performed according to the WHO protocols for each season [16].

From the 2021/2022 season onward, the Food and Drug Administration-cleared Panther Fusion™ SARS-CoV-2/Flu A/B/RSV system (Hologic, Inc.) was adopted.

Positive samples for IAVs or IBVs were further tested by in-house Real-Time PCR to determine the IAVs subtype or the IBVs lineage using primers and probe sequences according to WHO protocols.

### Statistical analysis

Influenza seasons are defined from week 46 of a given year to week 17 of the following year. Data were divided into the following groups to analyze influenza infection across different age groups: < 2, 2–4, 5–14, 15–44, 45–64, and ≥ 65 years, as per the ISS protocol. For each IAV subtype and IVB lineage we analyzed trends in peak-time and epidemic duration over the years. Each week, number of IAV samples that were not further tested were assigned to either A/H1N1pdm09 or A/H3N2 according to the respective proportions among the tested IAVs [17]. Similarly, number of IBV samples of unknown lineage were distributed between B/Victoria and B/Yamagata according to the proportions recovered on samples that went further testing. For each season and each subtype/lineage, we applied a 3 week moving average to the incidence timeseries and defined the peak as the week with maximum number of cases. We then defined the epidemic duration as the length of the briefest continuous period during which incidence exceeded 75% of the season’s total [18]. Peak week and epidemic duration were computed whenever at least 10 positive samples were collected for the subtype/lineage during the season. To analyze the impact of the COVID-19 pandemic we defined three periods: pre-pandemic (seasons 2017–2018, 2018–2019, 2019–2020), pandemic (2020–2021, 2021–2022), and post-pandemic (2022–2023, 2023–2024). We used the Mann–Whitney *U* test to compare pre- and post-pandemic peak and durations, considering separately IAV subtypes and IAV lineages, as the two IV species have typically different epidemic characteristics—e.g. IVB epidemics occurs usually later then IAV epidemics [17]. As a robustness check, we explored alternative window lengths and weighting functions for the moving average.

All data were analyzed using Microsoft Excel (http://www.microsoft.com, RRID:SCR_016137), and Python3, version 3.9.6, Scipy.stats package.

## Results

### Influenza confirmed cases

During the flu seasons from 2017/2018 to 2023/2024, 21,180 respiratory samples were analyzed (Table 1). The lowest number of samples (570 and 851) were collected in the 2020/2021 and 2021/2022 seasons, respectively, while the highest number (6,718) was examined in the 2023/2024 season. Of the 21,180 samples tested, 4,325 (20.42%) were positive for influenza. The proportion of influenza-positive cases ranged from 14.86% in the 2019/2020 season to 34.55% in 2021/2022. No positive influenza samples were detected during the 2020/2021 season, characterized by the COVID-19 pandemic. In the 2022/2023 season, activity returned to pre-pandemic levels with 3,866 samples collected, of which 830 (21.47%) were positive for influenza viruses. The number of samples increased in the following season to 6,718, with 1,220 (18.16%) testing positive for influenza viruses. Overall, IAVs accounted for 3,403 (78.68%) of all confirmed cases, more than 65% of cases in every season, except 2017/2018, when only 194 (26.72%) samples were IAVs-positives (Table 1). IBVs accounted for 922 (21.32%) of all confirmed cases, representing at least 10% of cases each season except 2018/2019 (0.11%) and 2021/2022 (no cases). The highest proportion of IBV-positive samples (532, 73.28%) was observed in the 2017/2018 season.

**Table 1:** Number of tested samples and percentage of positivity for influenza viruses, influenza A (IAV) and influenza B (IBV) viruses by season

Season	Number of samples	Influenza-positive		IAV-positive		IBV-positive	
		*n*	%	*n*	%†	*n*	%†
2017/2018	3,136	726	23.15	194	26.72	532	73.28
2018/2019	3,555	886	24.92	885	99.89	1	0.11
2019/2020	2,484	369	14.86	250	67.75	119	32.25
2020/2021	570	0	/	0	/	0	/
2021/2022	851	294	34.55	294	100	0	0
2022/2023	3,866	830	21.47	745	89.76	85	10.24
2023/2024	6,718	1,220	18.16	1,035	84.84	185	15.16
All-season	21,180	4,325	20.42	3,403	78.68	922	21.32

^†^Proportion of influenza-positive cases

### Analysis of influenza cases by season

**Fig. 1:**
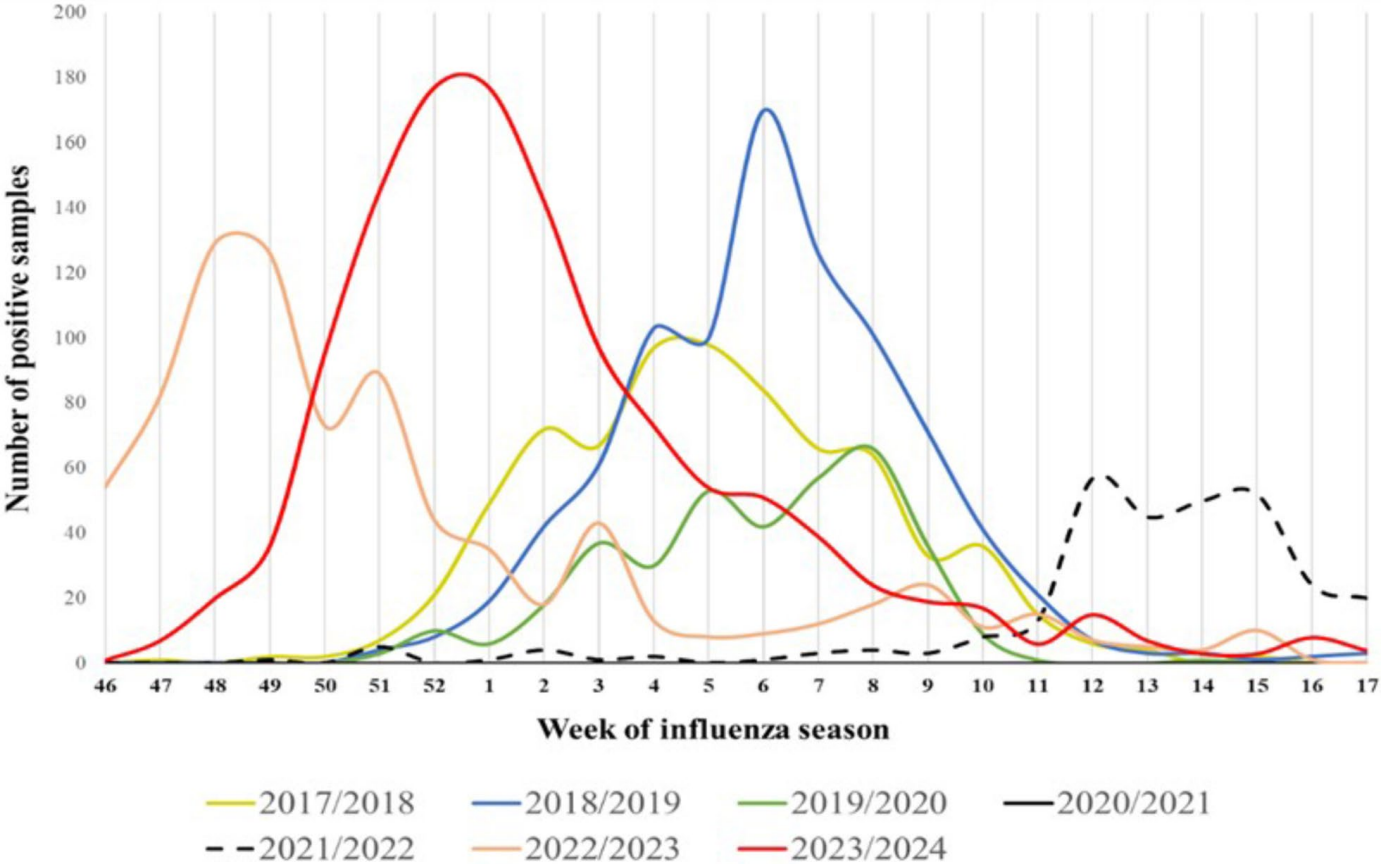
Distribution of influenza-positive samples in the Veneto Region by week

The weekly distribution of confirmed influenza cases (Fig. 1) followed the typical seasonal pattern until the 2019/2020 season, with a peak around the 5th–6th week. Due to the SARS-CoV-2 pandemic, a few samples (570) were collected during the 2020/2021 season, and all were negative. In the 2021/2022 season, samples increased to 851, reaching the highest positivity rate (294, 34.55%) from the 12th to the 16th week.

**Fig. 2:**
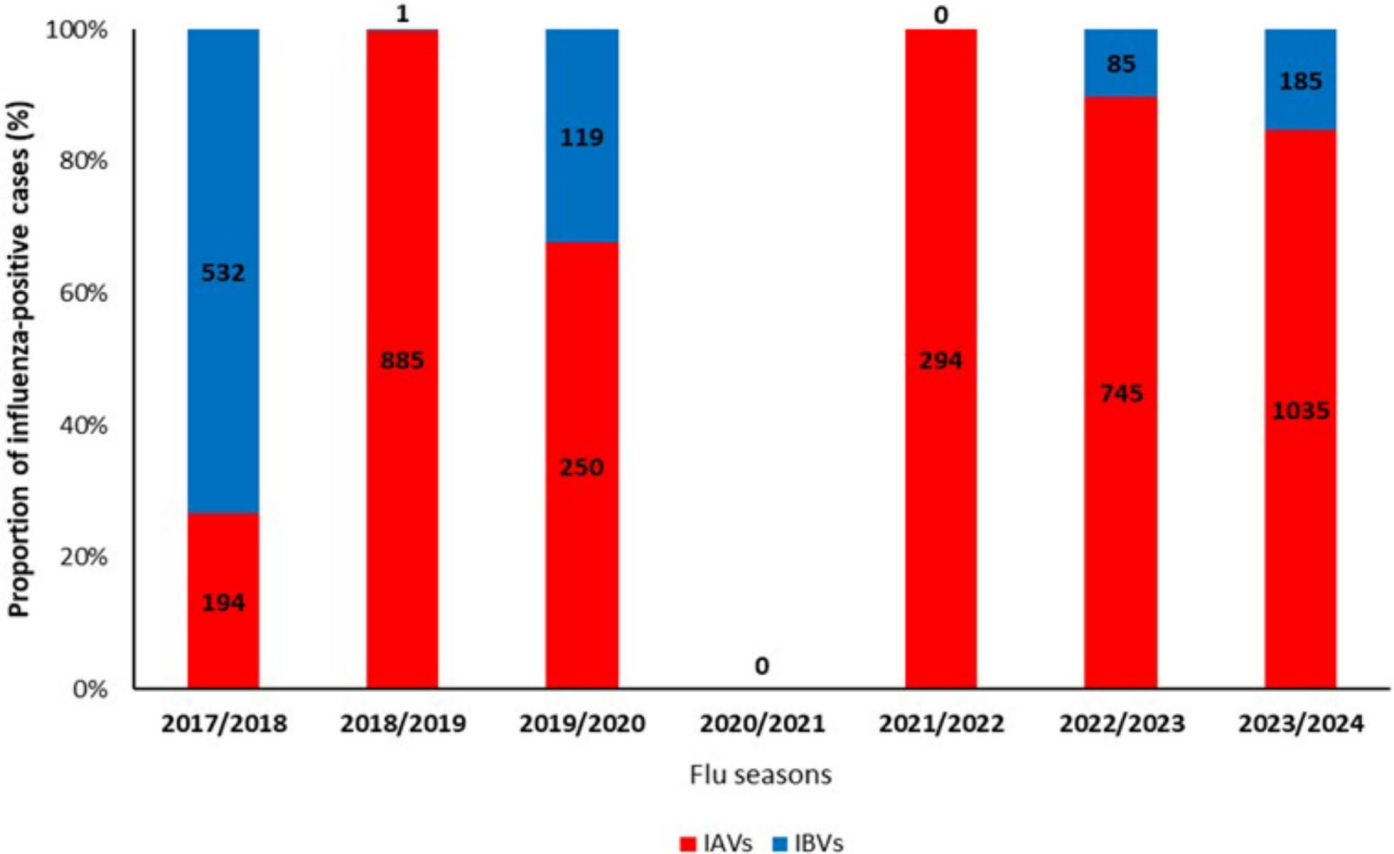
Relative percentage (y-axis) and numbers (on columns) of cases positive for influenza A viruses (IAVs) and B viruses (IBVs) by season

Regarding the proportion of influenza-positive cases, the flu seasons analyzed in this study are characterized by the predominance or the exclusive presence of IAVs, except for the 2017/2018 (Fig. 2).

### Circulation of IAV subtypes and IBV lineages

Data regarding IAV subtypes and IBV lineages identified during the 2017–2024 seasons are shown in Table 2; not all influenza-positive samples were characterized due to low viral load in the sample. During the 2017/2018 season, 681 of 726 influenza-positive samples were molecularly characterized: 507 (74.45%) were positive for IBVs and 174 (25.55%) for IAVs. Of IBV positive sample, 506 (74.30%) were IBV Yamagata lineage and one (0.15%) was IBV Victoria lineage. Regarding IAV positive samples, 152 (22.32%) were A(H1N1)pdm09, and 22 (3.23%) were A(H3N2). Only one coinfection between the A(H1N1)pdm09 and the IBV Yamagata was detected.

**Table 2:** A(H1N1)pdm09, A(H3N2), B/Victoria, and B/Yamagata cases detected during each influenza season. † The number of samples characterized is less than influenza positive samples due to low viral load in the sample

Season	Influenza positive samples	Samples characterized†	A(H1N1)pdm09		A(H3N2)		B/Victoria		B/Yamagata	
			*n*	%	*n*	%	*n*	%	*n*	%
2017/2018	726	681	152	22.32	22	3.23	1	0.15	506	74.30
2018/2019	886	777	372	47.88	404	51.99	1	0.13	0	0
2019/2020	369	307	68	22.15	140	45.60	99	32.25	0	0
2021/2022	294	283	3	1.06	280	98.94	0	0	0	0
2022/2023	830	736	80	10.87	579	78.67	77	10.46	0	0
2023/2024	1,220	877	729	83.12	29	3.31	119	13.57	0	0

In the 2018/2019 season, IAV A(H1N1)pdm09 was detected in 372 samples (47.88%) and A(H3N2) in 404 samples (51.99%); three cases of coinfection were observed. Only one sample of this season was positive for IBV (B/Victoria lineage).

The 2019/2020 season was characterized by limited surveillance activity. Of 307 samples, 68 (22.15%) were positive for A(H1N1)pdm09, 140 (45.60%) for A(H3N2), and 99 (32.25%) samples were positive for influenza B/Victoria. Interestingly, in the short period of surveillance, seven cases of coinfections were detected (4 A(H3N2)-B/Victoria, 2 A(H1N1)pdm09-B/Victoria, and 1 A(H1N1)pdm09-A(H3N2)).

As reported above, no influenza viruses were detected during the 2020/2021 season.

In the next season, 283 IAV positive samples were characterized, 3 (1.06%) were A(H1N1)pdm09, and 280 (98.94%) were A(H3N2) subtypes.

During the 2022/2023 season, 736 of the 830 positive samples were characterized: 80 (10.87%) A(H1N1)pdm09, 579 (78.67%) A(H3N2), and 77 (10.46%) B/Victoria. One sample tested positive for the B/Yamagata lineage; however, further investigations demonstrated that it resulted from a vaccine-related influenza virus B infection in a child with undiagnosed B-cell acute lymphoblastic leukemia [19].

In the 2023/2024 season, 877 of the 1,220 samples were further characterized: 729 (83.12%) A(H1N1)pdm09, 29 (3.31%) A(H3N2), and 119 (13.57%) B/Victoria.

Overall, the 2017/2018 season was dominated by the circulation of the B/Yamagata lineage, which was not observed in the following seasons. In contrast, the 2018/2019 season was dominated by IAV circulation, with both A(H1N1)pdm09 and A(H3N2) subtypes co-circulation. IAVs remained the prevalent type in the following seasons, with A(H3N2) being the dominant subtype from 2019/2020 to 2022/2023, while in the 2023/2024 season, A(H1N1)pdm09 became the prevalent subtype. Interestingly, no coinfections of influenza viruses were detected after the COVID-19 pandemic.

**Fig. 3:**
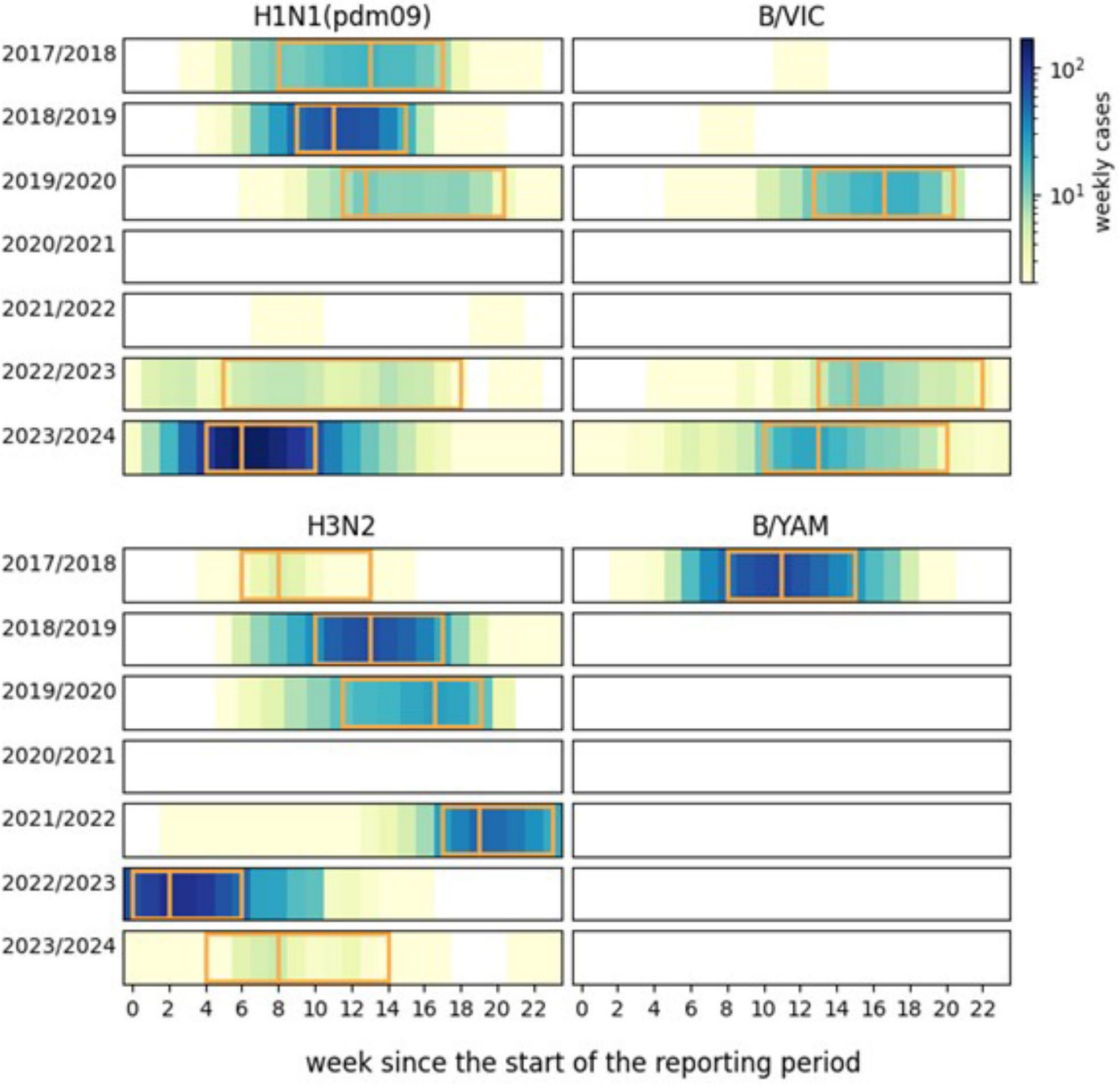
Weekly number of samples for each subtype/lineage and each season (color scale on thr top right). Data are smoothed with a 3-week moving average. For the cases in which at least 10 cumulative cases were detected during the season, the epidemic’s duration and peak are shown with the orange rectangle and the orange vertical lines respectively. The epidemic peak was not computed for the H1N1(pdm09) epidemic of 2021/2022 as the incidence profile was flat during the season

The 2022/2023 and 2023/2024 seasons showed earlier (46th and 49th week, respectively) and prolonged virus circulation (until the 9th of 2023 and the 10th week of 2024) compared to previous seasons. Stratifying by subtype/lineage we found a heterogenous behavior (Fig. 3). For the IAV A(H1N1)pdm09 epidemic of 2022/2023 we could not compute the peak week since no clear epidemic trend was observed, and the week with maximum incidence varied greatly according to the smoothing window used. Analyzing the other epidemics, we found that, for the IAV subtypes, the epidemic peaks occurred earlier in the post-pandemic, compared with the pre-pandemic period (Mann–Whitney *U* test, $$p=0.024)$$. The same was not true for the B lineages (Fig. 3, no test was performed due to the limited size of the sample). Finally, we find no significant change in epidemic duration from the pre- to the post-pandemic periods for both IAV and IBV.

### Distribution of influenza-positive samples in different age groups

In accordance with the RespiVirNet protocol, the positive samples were stratified by age groups: < 2, 2–4, 5–14, 15–44, 45–64, and ≥ 65 years old (Table 3). Unlike children under 14 years old, adults, especially those aged ≥ 65, experienced a more significant circulation of influenza viruses, reaching almost 50% of all cases in the 2019/2020 and 61.69% in the 2020/2021 flu seasons. This distribution pattern was not observed in the 2021/2022 season, where most cases were reported in the 15–44 years group, and only 10.20% were in the ≥ 65 age group.

**Table 3:** Number and percentage of positive samples for influenza virus, influenza A virus (IAV), and influenza B virus (IBV) in different age groups

Season	Category	IAV-positive		IBV-positive	
		*n*	%	*n*	%
2017/2018	Samples tested	194		532	
	Age groups (y)				
	< 2	27	13.92	17	3.20
	2 – 4	26	13.40	18	3.38
	5 – 14	12	6.19	18	3.38
	15 – 44	22	11.34	36	6.77
	45 – 64	42	21.65	125	23.50
	≥ 65	65	33.51	318	59.77
2018/2019	Samples tested	885		1	
	Age groups (y)				
	< 2	48	5.42	0	0
	2 – 4	22	2.49	0	0
	5 – 14	27	3.05	0	0
	15 – 44	76	8.59	1	100
	45 – 64	166	18.76	0	0
	> 65	546	61.69	0	0
2019/2020	Samples tested	250		119	
	Age groups (y)				
	< 2	19	7.60	13	10.92
	2 – 4	17	6.80	20	16.81
	5 – 14	18	7.20	33	27.73
	15 – 44	35	14.00	32	26.89
	45 – 64	39	15.60	5	4.20
	> 65	122	48.80	16	13.45
2021/2022	Samples tested	294		0	
	Age groups (y)				
	< 2	18	6.12		
	2 – 4	60	20.41		
	5 – 14	58	19.73		
	15 – 44	101	34.35		
	45 – 64	27	9.18		
	> 65	30	10.20		
2022/2023	Samples tested	745		85	
	Age groups (y)				
	< 2	90	12.08	11	12.94
	2 – 4	91	12.21	8	9.41
	5 – 14	134	17.99	25	29.41
	15 – 44	142	19.06	34	40.00
	45 – 64	111	14.90	6	7.06
	> 65	177	23.76	1	1.18
2023/2024	Samples tested	1,035		185	
	Age groups (y)				
	< 2	95	9.18	27	14.59
	2 – 4	109	10.53	23	12.43
	5 – 14	115	11.11	89	48.11
	15 – 44	91	8.79	29	15.68
	45 – 64	260	25.12	8	4.32
	> 65	365	35.27	9	4.86

## Discussion

Influenza is a severe public health problem due to the high number of hospitalizations and deaths. Most influenza-associated deaths occur among people aged 65 and older. Although the disease can be prevented with vaccination, prevention programs are below the desirable threshold due to vaccine hesitancy and no-vax theory. Additionally, the antigenic variations of influenza viruses pose a significant obstacle for the immunization of the population, reducing confidence in vaccination practices [11].

The WHO emphasizes the need for enhanced surveillance and adequate laboratory capacity to plan control strategies, enable early diagnosis, and respond to potential new variants and emerging influenza viruses from wildlife. Indeed, reports generated by surveillance systems and laboratories provide critical information for developing effective countermeasures, such as raising awareness among risk groups and the general population about vaccination campaigns, enhancing preparedness of the national health system network, updating diagnostic systems, controlling the spread and the severity of epidemic episodes, and minimizing the impact of potential influenza virus pandemics.

In Italy, the RespiVirNet system currently collects surveillance data on the circulation of different respiratory viruses from every Region. In the Veneto Region, the Microbiology and Virology Unit of the Hospital-University of Padua is the Regional Laboratory of Reference.

In the 2017/2018 season, 3,136 samples were processed, with 23.15% samples positive for influenza viruses, particularly IBVs. Confirmed cases of influenza were reported from the 49th week of 2017 until the 11th week of 2018, with the disease trend peaking around the 45th week of 2018. Data indicated that the B/Yamagata lineage was the most prevalent influenza virus, detected in more than 70% of samples. The same trend was observed nationally and in Europe [20]. Typically, flu seasons are characterized by the predominant circulation of IAVs due to their high antigenic variability, which gives them a significant ability to adapt to the host [11]. The high B/Yamagata lineage circulation in this season was probably due to the use of trivalent vaccines lacking this lineage [21]. However, in the rest of the world, there were no substantial differences between IAVs and IBVs frequency, or type A predominated, as reported in the Americas and the African Regions. This different circulation among the countries might be due to the intrinsic characteristics of the populations and environmental factors favoring the circulation of one type of flu over another [22]. Considering the high circulation of B/Yamagata in many areas of the world, the WHO subsequently proposed the quadrivalent vaccine formulation for the following seasons [23].

The 2018/2019 flu season was characterized by a cocirculation of the two IAV subtypes A(H1N1)pdm09 and A(H3N2), 47.88% and 51.99%, respectively, analyzing 3,555 samples. In this season the peak of influenza-positive cases was observed between the 4th and 9th weeks of 2019. Only one case of IBV, of the B/Victoria lineage, was identified in this flu season. Our data were in accord with the European data published in the ECDC’s annual report for the 2018/2019 flu season that highlighted the more significant circulation of IAVs (99%) with the cocirculation of A(H1N1)pdm09 (55%) and A(H3N2) (45%) subtypes. However, in Europe was also observed the circulation of IBV, mainly the B/Yamagata lineage then the B/Victoria lineage (79% vs 21% considering the IBV cases) [24].

The 2019/2020 flu season was characterized by low influenza virus circulation, detected in 14.86% of the 2,484 samples analyzed, the lowest positivity rate among the seasons considered in the Veneto Region in the last 7 years. Of these, 67.75% were reported as IAVs, with A(H3N2) accounting for 45.60% of all influenza viruses. The subtype A(H1N1)pdm09 was found in 22.15% of the cases compared to all influenza viruses. The circulation of IBVs (represented by Victoria lineage) was lower than IAVs, contributing to 32.25% of influenza-positive samples. From the 10th week of 2020, there was a drastic reduction in the incidence curve. Around this week, the surveillance system was suspended due to the rapid and widespread diffusion of SARS-CoV-2 in Italy and in particular in the Veneto Region [25]. The last surveillance report of the 2019/2020 season explicitly stated that the virological data contribution from regional laboratories was progressively reduced due to the COVID-19 emergency. Therefore, the published data did not reflect the real circulation of influenza viruses in Italy [26].

The 2020/2021 flu season occurred during the SARS-CoV-2 pandemic, which significantly impacted influenza virus circulation. Only 570 samples were analyzed in Italy, and none tested positive for influenza. This absence of influenza cases was observed across the country and may be due to pandemic-related measures like lockdowns, mask-wearing, sanitization [27] or viral interference. The European Centre for Disease Prevention and Control (ECDC) described the 2020/2021 flu season as unusual, declaring influenza virus activity very low; reports of positive cases involved only a few countries with rare hospitalization cases. A strong decline of influenza circulation was also reported outside Europe, worldwide [17, 28].

In the 2021/2022 flu season, unlike the previous flu season, several positive cases of influenza virus were reported in the Veneto Region. Overall, 851 samples were analyzed, with most positive cases occurring between the twelfth and sixteenth weeks of 2022. During the 2021/2022 season, only IAVs circulated; of these, 98.94% were A(H3N2), and 1.06% were A(H1N1)pdm09. This is according to the European situation, as shown in the ECDC’s annual epidemiological report for the 2021/2022 flu surveillance season, where 98.70% of positive samples were identified as IAVs and only 1.30% as IBVs. The seasonal trend of the incidence curve was also comparable, with the peak around the 13th week.

During the 2022/2023 flu season, 3,866 samples were analyzed, with an influenza positivity rate of 21.47%, of which 89.76% were IAVs. The most common IAV subtype was A(H3N2), accounting for 78.67% of all positive influenza virus cases, whereas A(H1N1)pdm09 was reported in 10.87% of cases. All positive IBV samples belonged to the Victoria lineage. During this season, influenza virus circulation returned to almost pre-pandemic levels. Unlike the previous flu seasons, the seasonal epidemic started earlier, and the peak of incidence was reached around the 48th week of 2022. Additionally, this flu season shows an extended period of influenza virus circulation compared to previous seasons. These was due to the combination of two waves one after the other. IAVs were predominant in the early phase of the epidemic, but in the later phase of the season, almost all reported influenza virus infections were associated with IBV, showing a mutually exclusive circulation.

The 2023/2024 flu season was characterized by the highest number of samples tested. Among the 6,718 samples, 18.16% were positive for influenza viruses, which peaked between the last week of 2023 and the first week of 2024. The most prevalent influenza virus was IAVs (84.84%), with 83.12% of A(H1N1)pdm09 and 3.31% of A(H3N2). Interestingly, compared to the previous influenza season, there was an inversion of the predominant circulating strain from A(H3N2) to A(H1N1)pdm09 while maintaining a cocirculation of IAVs and IBVs with a later increase in IBV cases. The reversal in the trend of the circulating strain could be explained by the fact that the prolonged circulation of the A(H3N2) virus in previous years has contributed to increasing population immunity towards this subtype. This has made its spread more difficult compared to (H1N1)pdm09, to which the population was more susceptible. We may expect vaccination does not have a strong impact on the type of circulating strain, given the low vaccine adherence in both the at-risk and general populations.

Notably, the increased number of samples tested for influenza reflects the different diagnostic approaches. In the seasons from 2017 to 2020, influenza viruses were tested in individuals with influenza-like illnesses and suspected influenza etiology. From 2021 onwards, the subjects analyzed included those with suspected SARS-CoV-2 and RSV etiology. This change may bias the positivity rates, affecting the direct comparison of positivity rates between the 2017–2020 and 2021–2024 seasons. However, it is possible to analyze the weekly positivity rate and the peak for each flu season. In fact, in the post-pandemic seasons from 2021 to 2023, the peak of cases no longer occurred from the second to the third weeks of the year. In the 2021/2022 season, it was delayed following the reduction of influenza virus circulation due to pandemic restrictions, whereas in the 2022/2023 season started even before the seasonal surveillance began, probably due to the immunity gap in the population, and in the last season, it was around the end of the 2023 and the beginning of 2024.

Regarding the influenza virus type, IAVs are generally the most represented and widespread due to their high genetic variability and, therefore, a more remarkable ability to counteract host defenses than IBVs [13]. The alternating circulation of IBV lineages compared to IAVs, which is particularly evident in the last two seasons, may be imputed to viral interference, as well as the immune memory developed by the patients following previous IBV infections, which provides some, albeit incomplete, immune coverage for the following year against the same virus type.

Analyzing the distribution of influenza-positive samples across age groups, there is generally a direct proportionality between increasing age and positivity for IAVs. However, this trend is not evident in the 2021/2022 season, where subjects over 45 had a lower positivity rate than those aged 2 to 44 years (9.69% vs. 24.83%), with a peak in the 15 to 44-year-old group. This season was characterized by pandemic restrictions, which might suggest that subjects over 45 years old were more compliant with restrictive measures and were more effectively targeted by the flu vaccination campaign. Actually, this season was characterized by the highest vaccination coverage for influenza [29]. In contrast, the 15-to 44-year-old age group in the 2021/2022 season showed the highest positivity rate among the three seasons considered (34.35%). This could be attributed to a lack of preventive measures, an immunity gap resulting from the lack of contact with influenza viruses in the previous season, and low adherence to influenza vaccination in this age group [30]. In fact, influenza vaccination in Italy exhibits notably low rates when considering the general population (range 15.3–23.7% across the influenza seasons analyzed). In age groups below 60 years, the vaccination rate is less than 10% (between 3 and 9%), increasing to approximately 20% among individuals aged 60–64 years, and exceeding 50% in subjects aged 65 years and older (with variations between 52.7% and 65.3% across the influenza seasons studied) 29].

Regarding IBVs, in the 2017/2018 season, dominated by the B/Yamagata lineage, the positivity rate increased with age. In other seasons, where only the B/Victoria lineage circulated, most positive cases were among those aged 5 to 14. Many studies confirm our observation: the B/Yamagata lineage preferentially infects adults and older people, while the B/Victoria lineage is more common in those up to 15 years old [31–33]. Since the 2017/2018 influenza season, no cases related to the IBV Yamagata lineage have been reported in the Veneto Region. Additionally, since March 2020, the IBV Yamagata lineage has not been reported globally, suggesting the possible extinction of the virus [34]. The extinction of the B/Yamagata lineage was driven by three main factors: preventive measures during the pandemic, reduced circulation due to decreased travel, and the intrinsic genetic characteristics of the virus. Given the theoretical risk of reintroducing this virus into the population through live attenuated vaccines containing the IBV Yamagata lineage, the WHO recommends its exclusion in the vaccine formulation [35].

This study may be subject to some limitations due to the variability in the number of samples analyzed across different seasons, particularly coinciding with the initial phases of the COVID-19 pandemic, which could create biases in the obtained results. Furthermore, there is also a risk of having underestimated influenza cases during the COVID-19 pandemic when activities were polarized towards the diagnosis and surveillance of SARS-CoV-2 infections. This could affect the reported positivity rates for influenza viruses. However, the fact that the results obtained in the Veneto Region are in line with national data suggests that the trend in the circulation of influenza viruses described in this work has not been significantly influenced.

## Conclusion

Data on influenza surveillance in the Veneto Region from 2017 to 2024 overlap the trends observed in the rest of Italy. Results support four key concepts: the interference of SARS-CoV-2 circulation on influenza virus circulation, the immunity gap in the population caused by SARS-CoV-2 pandemic restrictions, the extinction of the B/Yamagata lineage, and the varying susceptibility of the population to influenza infection depending on the age group considered.

## Data Availability

Data are available upon motivated request.

## References

[1] Tran K, Cimon K, Severn M, Pessoa-Silva CL, Conly J. Aerosol generating procedures and risk of transmission of acute respiratory infections to healthcare workers: a systematic review. PLoS ONE. 2012;7:e35797. 22563403 10.1371/journal.pone.0035797 PMC3338532

[2] Otter JA, Donskey C, Yezli S, Douthwaite S, Goldenberg SD, Weber DJ. Transmission of SARS and MERS coronaviruses and influenza virus in healthcare settings: the possible role of dry surface contamination. J Hosp Infect. 2016;92:235–50. 26597631 10.1016/j.jhin.2015.08.027 PMC7114921

[3] Leung NHL. Transmissibility and transmission of respiratory viruses. Nat Rev Microbiol. 2021;19:528–45. 33753932 10.1038/s41579-021-00535-6 PMC7982882

[4] Thompson WW, Weintraub E, Dhankhar P, et al. Estimates of US influenza-associated deaths made using four different methods. Influenza Other Respir Viruses. 2009;3:37–49. 19453440 10.1111/j.1750-2659.2009.00073.x PMC4986622

[5] Nair H, Brooks WA, Katz M, et al. Global burden of respiratory infections due to seasonal influenza in young children: a systematic review and meta-analysis. Lancet. 2011;378:1917–30. 22078723 10.1016/S0140-6736(11)61051-9

[6] Krammer F, Smith GJD, Fouchier RAM, et al. Influenza. Nat Rev Dis Primers. 2018;4:3. 29955068 10.1038/s41572-018-0002-y PMC7097467

[7] Nypaver C, Dehlinger C, Carter C. Influenza and influenza vaccine: a review. J Midwifery Womens Health. 2021;66:45–53. 33522695 10.1111/jmwh.13203 PMC8014756

[8] Świerczyńska M, Mirowska-Guzel DM, Pindelska E. Antiviral drugs in influenza. Int J Environ Res Public Health. 2022;19:3018. 35270708 10.3390/ijerph19053018 PMC8910682

[9] Kumari R, Sharma SD, Kumar A, et al. Antiviral approaches against influenza virus. Clin Microbiol Rev. 2023;36:e00040. 36645300 10.1128/cmr.00040-22 PMC10035319

[10] Sukhdeo S, Lee N. Influenza: clinical aspects, diagnosis, and treatment. Curr Opin Pulm Med. 2022;28:199–204. 35125406 10.1097/MCP.0000000000000860

[11] Smyk JM, Szydłowska N, Szulc W, Majewska A. Evolution of influenza viruses—drug resistance, treatment options, and prospects. Int J Mol Sci. 2022;23:12244. 36293099 10.3390/ijms232012244 PMC9602850

[12] Biere B, Bauer B, Schweiger B. Differentiation of influenza B virus lineages yamagata and victoria by real-time PCR. J Clin Microbiol. 2010;48:1425–7. 20107085 10.1128/JCM.02116-09 PMC2849545

[13] Petrova VN, Russell CA. The evolution of seasonal influenza viruses. Nat Rev Microbiol. 2018;16:47–60. 29109554 10.1038/nrmicro.2017.146

[14] AbuBakar U, Amrani L, Kamarulzaman FA, Karsani SA, Hassandarvish P, Khairat JE. Avian influenza virus tropism in humans. Viruses. 2023;15:833. 37112812 10.3390/v15040833 PMC10142937

[15] German RR, Lee LM, Horan JM, et al. Updated guidelines for evaluating public health surveillance systems: recommendations from the Guidelines Working Group. MMWR Recomm Rep 2001;50:1–35; quiz CE1–7. 18634202

[16] World Health Organization (WHO). WHO information for the molecular detection of influenza viruses [Internet]. 2021. Available from: https://cdn.who.int/media/docs/default-source/influenza/molecular-detention-of-influenza-viruses/protocols_influenza_virus_detection_feb_2021.pdf

[17] Finkelman BS, Viboud C, Koelle K, Ferrari MJ, Bharti N, Grenfell BT. Global patterns in seasonal activity of influenza A/H3N2, A/H1N1, and B from 1997 to 2005: viral coexistence and latitudinal gradients. PLoS ONE. 2007;2:e1296. 18074020 10.1371/journal.pone.0001296 PMC2117904

[18] Del Riccio M, Caini S, Bonaccorsi G, Lorini C, Paget J, van der Velden K, et al. Global analysis of respiratory viral circulation and timing of epidemics in the pre–COVID-19 and COVID-19 pandemic eras, based on data from the Global Influenza Surveillance and Response System (GISRS). Int J Infect Dis. 2024;144:107052. 38636684 10.1016/j.ijid.2024.107052

[19] Di Pietra G, Di Sopra S, Conciatori V, et al. Vaccine-related influenza virus B infection in a child with an undiagnosed B-cell acute lymphoblastic leukemia. Int J Infect Dis. 2024;147:107184. 39033799 10.1016/j.ijid.2024.107184

[20] Adlhoch C, Snacken R, Melidou A, Ionescu S, Penttinen P. European influenza surveillance network. Dominant influenza A(H3N2) and B/Yamagata virus circulation in EU/EEA, 2016/17 and 2017/18 seasons, respectively. Euro Surveill. 2018;23:18–00146. 29616611 10.2807/1560-7917.ES.2018.23.13.18-00146 PMC5883452

[21] Loconsole D, De Robertis AL, Morea A, et al. High public-health impact in an influenza-B-mismatch season in Southern Italy, 2017–2018. Biomed Res Int. 2019;2019:4643260. 31531353 10.1155/2019/4643260 PMC6720359

[22] Lina B, Georges A, Burtseva E, et al. Complicated hospitalization due to influenza: results from the global hospital influenza network for the 2017–2018 season. BMC Infect Dis. 2020;20:465. 32615985 10.1186/s12879-020-05167-4 PMC7330273

[23] Drori Y, Pando R, Sefty H, et al. Influenza vaccine effectiveness against laboratory-confirmed influenza in a vaccine-mismatched influenza B-dominant season. Vaccine. 2020;38:8387–95. 33243633 10.1016/j.vaccine.2020.10.074

[24] European Centre for Disease Prevention and Control. Seasonal influenza 2018–2019. https://www.ecdc.europa.eu/sites/default/files/documents/AER_for_2018_seasonal-influenza-corrected.pdf Accessed October, 2019

[25] Lavezzo E, Franchin E, Ciavarella C, et al. Suppression of a SARS-CoV-2 outbreak in the Italian municipality of Vo’. Nature. 2020;584:425–9. 32604404 10.1038/s41586-020-2488-1 PMC7618354

[26] Istituto Superiore di Sanità. Rapporto InfluNet Virologico 17/2020. https://www.salute.gov.it/portale/temi/documenti/virologica/AggVir_29-04-20.pdf Accessed April, 2020

[27] Principi N, Autore G, Ramundo G, Esposito S. Epidemiology of respiratory infections during the COVID-19 pandemic. Viruses. 2023;15:1160. 37243246 10.3390/v15051160 PMC10224029

[28] Bonacina F, Boëlle P-Y, Colizza V, Lopez O, Thomas M, Poletto C. Global patterns and drivers of influenza decline during the COVID-19 pandemic. Int J Infect Dis. 2023;128:132–9. 36608787 10.1016/j.ijid.2022.12.042 PMC9809002

[29] Istituto Superiore di Sanità. Coperture della vaccinazione antinfluenzale in Italia. https://www.epicentro.iss.it/influenza/coperture-vaccinali Accessed July, 2023

[30] Ministero della Salute. Vaccinazione Antinfluenzale: 2021–2022 - Coperture Vaccinali per 100 Abitanti. https://www.salute.gov.it/imgs/C_17_tavole_19_1_22_file.pdf Accessed July, 2022

[31] Caini S, Kusznierz G, Garate VV, et al. The epidemiological signature of influenza B virus and its B/Victoria and B/Yamagata lineages in the 21st century. PLoS ONE. 2019;14:e0222381. 31513690 10.1371/journal.pone.0222381 PMC6742362

[32] Yang J, Lau YC, Wu P, et al. Variation in influenza B virus epidemiology by lineage. China Emerg Infect Dis. 2018;24:1536–40. 30015611 10.3201/eid2408.180063 PMC6056115

[33] Xu C, Chan K-H, Tsang TK, et al. Comparative epidemiology of influenza b yamagata- and victoria-lineage viruses in households. Am J Epidemiol. 2015;182:705–13. 26400854 10.1093/aje/kwv110 PMC4715237

[34] Paget J, Caini S, Del Riccio M, van Waarden W, Meijer A. Has influenza B/Yamagata become extinct and what implications might this have for quadrivalent influenza vaccines? Eurosurveillance. 2022;27(39):2200753. 36177871 10.2807/1560-7917.ES.2022.27.39.2200753 PMC9524051

[35] World Health Organization. Recommended Composition of Influenza Virus Vaccines for Use in the 2024–2025 Northern Hemisphere Influenza Season. https://cdn.who.int/media/docs/default-source/influenza/who-influenza-recommendations/vcm-northern-hemisphere-recommendation-2024-2025/recommended-composition-of-influenza-virus-vaccines-for-use-in-the-2024-2025-northern-hemisphere-influenza-season.pdf?sfvrsn=2e9d2194_7&download=true Accessed February, 2024

